# Lactic Acid Bacteria Isolated from Fresh Vegetable Products: Potential Probiotic and Postbiotic Characteristics Including Immunomodulatory Effects

**DOI:** 10.3390/microorganisms10020389

**Published:** 2022-02-08

**Authors:** Fatima Alameri, Mohammad Tarique, Tareq Osaili, Riyad Obaid, Abdelmoneim Abdalla, Razan Masad, Ashraf Al-Sbiei, Maria Fernandez-Cabezudo, Shao-Quan Liu, Basel Al-Ramadi, Mutamed Ayyash

**Affiliations:** 1 Department of Food Science, College of Agriculture and Veterinary Medicine, United Arab Emirates University (UAEU), Al Ain P.O. Box 15551, United Arab Emirates; 201870222@uaeu.ac.ae (F.A.); 201990207@uaeu.ac.ae (M.T.); 2 Department of Clinical Nutrition and Dietetics, College of Health Sciences, University of Sharjah, Sharjah P.O. Box 32223, United Arab Emirates; tosaili@sharjah.ac.ae (T.O.); robaid@sharjah.ac.ae (R.O.); 3 Department of Nutrition and Food Technology, Faculty of Agriculture, Jordan University of Science and Technology, P.O. Box 3030, Irbid 22110, Jordan; 4 Food Science Department, College of Agriculture, South Valley University, Qena 83523, Egypt; aabdalla7@gmail.com; 5 Department of Medical Microbiology and Immunology, College of Medicine and Health Sciences, United Arab Emirates University (UAEU), Al Ain P.O. Box 15551, United Arab Emirates; 201890026@uaeu.ac.ae (R.M.); ramadi.b@uaeu.ac.ae (B.A.-R.); 6 Department of Biochemistry & Molecular Biology, College of Medicine and Health Sciences, United Arab Emirates University (UAEU), Al Ain P.O. Box 15551, United Arab Emirates; 201180866@uaeu.ac.ae (A.A.-S.); mariac@uaeu.ac.ae (M.F.-C.); 7 Zayed Center for Health Sciences, United Arab Emirates University (UAEU), Al Ain P.O. Box 15551, United Arab Emirates; 8 Department of Food Science and Technology, Faculty of Science, National University of Singapore, Singapore 117542, Singapore; fstlsq@nus.edu.sg

**Keywords:** autoaggregation, antimicrobial, cholesterol-lowering, immunomodulation

## Abstract

The ability to perform effectively in the gastrointestinal tract (GIT) is one of the most significant criteria in the selection of potential probiotic bacteria. Thus, the present study aimed to investigate the potential probiotic characteristics of some selected lactic acid bacteria (LAB) isolated from vegetable products. Probiotic characteristics included tolerance to acid and bile, cholesterol-removing ability, bile salt hydrolysis, resistance against lysozyme and antibiotics, production of exopolysaccharides (EPS), antimicrobial and hemolytic activities, and cell surface characteristics (auto-aggregation, co-aggregation, and hydrophobicity). The survival rate of isolates after G120 ranged from 8.0 to 8.6 Log_10_ CFU/mL. After the intestinal phase (IN-120), the bacterial count ranged from 7.3 to 8.5 Log_10_ CFU/mL. The bile tolerance rates ranged from 17.8 to 51.1%, 33.6 to 63.9%, and 55.9 to 72.5% for cholic acid, oxgall, and taurocholic acid, respectively. Isolates F1, F8, F23, and F37 were able to reduce cholesterol (>30%) from the broth. The auto-aggregation average rate increased significantly after 24 h for all isolates, while two isolates showed the highest hydrophobicity values. Moreover, isolates had attachment capabilities comparable to those of HT-29 cells, with an average of 8.03 Log_10_ CFU/mL after 2 h. All isolates were resistant to lysozyme and vancomycin, and 8 out of the 17 selected isolates displayed an ability to produce exopolysaccharides (EPS). Based on 16S rRNA sequencing, LAB isolates were identified as *Enterococcus faecium*, *E. durans*, *E. lactis*, and *Pediococcus acidilactici*.

## 1. Introduction

The gut microbiota have the capacity to interact with human cells, including specific immune cells. These interactions yield different health benefits in the host, including regulation of GIT motility, destroying toxins and mutagens, transforming bile acid and steroids, producing vitamins, absorbing minerals, and modulating mucosal and systemic immunity. Lactic acid bacteria, possessing potential probiotic characteristics and isolated from fermented food products, play a crucial role in improving the quality of the gut microbiota [1,2]. Probiotics are defined as “live microorganisms which, when administered in adequate amounts, confer a health benefit on the host” [3]. Based on this definition, a microorganism is labeled as probiotic only when there is scientific evidence proving its potential health benefits to the host [4]. The International Scientific Association for Probiotics and Prebiotics (ISAPP) states that for a microorganism to be described as probiotic, it should first go through a series of human or intended user trials to ensure safety and to prove at least one of the health benefits that the microorganism is claimed to provide for the host [3]. In general, the most common microorganisms added to food products or supplements for their probiotic abilities include members of the lactic acid bacteria (LAB) and bifidobacteria [1]. The nonviable (killed/dead) cells of probiotics have also been reported to exhibit health benefits [5,6]. This encouraged the ISAPP to issue a consensus on the definition of postbiotics (nonviable cells) [7]. Thus, a postbiotic is defined as a “preparation of inanimate microorganisms and/or their components that confers a health benefit on the host” [7].

The capacity of bacteria to survive through the GI system, to reach either the small or large intestine in sufficient numbers, and to interact with and/or attach to and colonize the host must be confirmed before probiotics can be added to foods and supplements for their possible health advantages [1]. Several factors have a deleterious effect on probiotics, including the stomach’s high acidity (pH 1.5–3.0), bile salts, and digestive enzymes. Additionally, prior to consumption, the probiotics must maintain viability throughout culture manufacture and storage, product or supplement manufacture, and product shelf-life [1].

Clinical studies have demonstrated various health effects associated with consumption of these microorganisms (live and killed), such as reduction in duration and occurrences of diarrhea, alleviation of symptoms of lactose intolerance, reduced incidences of pathogenic infection, and stimulation of the immune system and regulation of the inflammatory response [8,9]. The present study aimed to investigate the potential probiotic characteristics, i.e., tolerance to acid and bile, cholesterol removing ability, bile salt hydrolysis, resistance against lysozyme and antibiotics, production of exopolysaccharides (EPS), antimicrobial and hemolytic activities, and cell surface characteristics (auto-aggregation, co-aggregation and hydrophobicity), of some selected LAB isolated from vegetable products [1].

Fruits and vegetables are one of the main dietary requirements in the adult diet and, according to the Dietary Guidelines for Americans, it is recommended to consume half a plate of fruits or vegetables in all meals [10]. In terms of nutritional composition, fruits and vegetables are considered highly nutritious foods as they provide high amounts of vitamins, such as vitamin C and A; minerals, specifically electrolytes; and phytochemicals, specific antioxidants that fight free radicals [10]. Thus, the current study aimed to isolate LAB from fresh vegetables and characterize these isolates as potential probiotics according to different properties, including (1) gastrointestinal tolerance to (a) in vitro digestion, (b) bile salts, and (c) lysozyme; (2) physiological properties such as (a) auto-aggregation, (b) co-aggregation, (c) hydrophobicity, (d) adhesion to HT-29 cells, and (e) cholesterol reduction; (3) production of desirable substances such as (a) bile salt hydrolase, (b) antimicrobials, and (c) EPS; and (4) bioactivities such as immunomodulation and sensitivity to antibiotics.

## 2. Materials and Methods

### 2.1. Sample Collection

Samples (140) of fresh fruits and vegetables (various types, namely tomato, cucumber, strawberry, peach, lettuce, parsley, and cabbage) were collected from local markets (Sharjah, United Arab Emirates) and transported in an icebox to the food microbiology lab of the University of Sharjah, Sharjah, United Arab Emirates, where isolation was carried out on De Man, Rogosa, and Sharpe (MRS) agar. Characterization of the LAB isolates as potential probiotics was carried out in the food microbiology lab of United Arab Emirates University (UAEU). Unless otherwise mentioned, the isolation and characterization were completed using Sigma-Aldrich chemicals (St. Louis, MO, USA).

### 2.2. Isolation of Lactic Acid Bacteria

The pour-plate technique was performed using MRS agar, and the plates were incubated anaerobically at 37 °C for 24 h in a CO_2_ incubator (Binder C 170, Tuttlingen, Germany). The Gram-positive and catalase-negative isolates were subcultured in MRS broth, and then the working stocks were prepared using 50 mL:50 mL glycerol:water. The stocks were stored at −80 °C. Overnight activation at 37 °C was carried out to investigate the potential probiotic characteristics of the isolates.

### 2.3. Tolerance to Stimulated Digestion Conditions Using INFOGEST2.0

Isolates grown overnight were centrifuged, and then the pellets were resuspended in 1 mL of 0.1 mM sodium phosphate buffer (pH 7.0). The in vitro digestion was done as per Brodkorb [8]. Briefly, 1 mL of culture was mixed with 1 mL of oral stimulated salivary fluids (amylase 75 U/mL, salivary fluid SSF pH 7.0, 0.3 M CaCl_2_, total volume 2 mL) for 2 min. Afterward, the 2 mL of oral bolus was mixed with 2 mL simulated gastric fluids (pepsin 2000 U/mL, gastric lipase using rabbit gastric extract RGE 60 U/mL (Lipolytech, Marseille, France), stimulated gastric fluids (SGF) pH 3.0, 0.3 M CaCl_2_, total volume 4 mL) for 120 min at 37 °C. Finally, the 4 mL of gastric chyme was mixed with 4 mL stimulated intestinal fluids (pancreatin 100 U/mL, bile 10 mmol/L, stimulated intestinal fluids (SIF) pH 7.0, 0.3 M CaCl_2_, total volume 24 mL) for 120 min at 37 °C. After the digestion steps were completed, a 1 mL sample of the digest was taken aseptically and serial dilutions were made. The bacterial enumeration was carried out on MRS agar using the pour-plate technique. After anaerobic incubation at 37 °C for 48 h, the plates were counted using a colony counter (Interscience San 1200; New York, NY, USA).

### 2.4. Bile Tolerance

The bile tolerance test was carried out according to Liong and Shah [6] by adding cholic acid (0.30%), taurocholic acid (1.0%), and oxgall (1.0%) to the overnight-activated isolates in MRS broth at 37 °C. The absorbance was then measured at 620 nm after 0, 3, and 6 h using a microplate spectrophotometer (Epoch-2, BioTek; Winooski, VT, USA). The activated cultures in MRS broth without bile salt were employed as positive controls to calculate the percentage of growth according to the following equation:

$$\%\;\mathrm{of}\;\mathrm{Growth}\;=\frac{\mathrm{Growth}\;\mathrm{in}\;\mathrm{bile}\;\mathrm{broth}\;}{\mathrm{Growth}\;\mathrm{in}\;\mathrm{control}\;\mathrm{broth}}\times 100$$

### 2.5. Bile Salt Hydrolase (BSH) Activity

BSH activity was examined as described by Ayyash [11] by detecting the amino acids released from the conjugated bile salts (6 mM sodium glycochenodeoxycholic, taurocholic, taurochenodeoxycholic, and taurodeoxycholic acids) by measuring the absorbance of ninhydrin at 570 nm using an Epoch-2 Microplate Spectrophotometer (BioTek, Rancho Cordova, CA, USA).

### 2.6. Cholesterol Removal

The cholesterol removal ability of the isolates was determined as per Shivangi [12]. The activated cultures were inoculated in MRS broth supplemented with 100 µg/mL water-soluble cholesterol (Sigma, St. Louis, MI, USA), followed by incubation at 37 °C for 24 h. The uninoculated tube was employed as a control. After incubation, the cells were removed by centrifuging at 6000× *g* for 15 min. The spent broth was collected in clean, dry tubes. From the collected spent broth, 0.5 mL was placed in a test tube and 3 mL of 95% ethanol was added followed by 2 mL of 50% KOH. After mixing thoroughly, the contents were heated at 60 °C for 10 min in a water bath and subsequently cooled. To each tube, 5 mL of n-hexane was added and mixed, followed by a further 3 mL of distilled water which was also mixed. The tubes were allowed to stand for 15 min at room temperature to separate the phases. After phase separation, 2.5 mL of the upper hexane layer was separated and placed in a clean, dry test tube. The hexane was evaporated at 60 °C under the flow of nitrogen gas. To each tube, 4 mL of o-phthaldehyde reagent was added and allowed to stand at room temperature for 10 min. To each tube, 2 mL of concentrated sulfuric acid was added slowly to the side of tube, mixed, and allowed to stand for 10 min. The color developed was measured at 550 nm using an Epoch-2 Microplate Spectrophotometer (Agilent Technologies, Santa Clara, CA, USA).

### 2.7. Auto-Aggregation

Auto-aggregation of the activated cultures was determined according to Gao [13], and the absorbance was measured at 0, 4, and 24 h at 600 nm. Auto-aggregation was estimated according to the following equation:

$$\mathrm{Auto}-\mathrm{aggregation}\;(\%)=\left[1-\frac{A_{t}}{A_{0}}\right]\times 100$$

where A_t_ is absorbance at time t, and A_0_ is absorbance at time 0.

### 2.8. Hydrophobicity

Hydrophobicity of the isolates to various hydrocarbons, namely xylene, hexadecane, and octane, was evaluated according to Abushelaibi [14], and the final absorbance was measured at 600 nm using an Epoch-2 Microplate Spectrophotometer. The reduced absorbance in the aqueous phase was taken as measure of cell surface hydrophobicity. The hydrophobicity was calculated as per the following equation:

$$\mathrm{Hydrophobicity}\;(\%)=\left[\frac{A\;-A_{0}}{A}\right]\times 100$$

where A is the initial absorbance at 600 nm and A_0_ is the final absorbance.

### 2.9. Adhesion to HT-29 Cells

To determine the adhesion ability, HT-29 cells were seeded on to 24-well tissue culture plates at a concentration of 10^5^ cells/well and incubated at 37 °C for 24 h in a 5% CO_2_ and 95% air atmosphere. The isolate cells from 20 h cultures were washed twice with Dulbecco’s phosphate-buffered saline (DPBS) and then resuspended in nonsupplemented DMEM at a concentration of 10^7^–10^8^ CFU/mL. These bacterial suspensions were added to the monolayers of the HT-29 cells. After 2 h of incubation at 37 °C in a 5% CO_2_ and 95% air atmosphere, the wells were washed three times with PBS to remove the bacterial suspensions and nonadherent cells and treated with 1% Triton X-100 (Sigma–Aldrich, St. Louis, MO, USA) solution to detach the adherent bacteria. The final viable LAB cell counts (adherent LAB) were enumerated on MRS agar [15]. Adhesion percentage was then estimated using the following equation:

$$\mathrm{Adhesion}\;\mathrm{ability}\;(\%)=\left[\frac{A_{t}}{A_{0}}\right]\times 100$$

where A_t_ is the number of the adhered cells (log CFU/mL) after incubation, and A_0_ is the initial cell number (log CFU/mL).

### 2.10. Co-Aggregation

Co-aggregation was examined according to Abushelaibi [14] using four pathogenic bacteria, namely *Escherichia coli* 0157:H7 1934, *Staphylococcus aureus* ATCC 25923, *Salmonella* Typhimurium 02-8423, and *Listeria monocytogenes* DSM 20649. The results were expressed as co-aggregation percentages utilizing the following equation:

$$\mathrm{Co}-\mathrm{aggregation}\;(\%)=\left[1-\frac{A_{t}}{A_{0}}\right]\times 100\;$$

### 2.11. Antimicrobial Production

Antimicrobial activity was determined using a cell-free supernatant as per Ayyash [11]. Briefly, the MRS agar plates were overlaid with 7 mL of soft MRS agar inoculated with 20 µL of overnight-activated culture of indicator strains. Different wells were made in the agar. Wells were filled with 50 Al cell-free broth of 24 h old cultures obtained by centrifuging the culture broth at 5000 rpm for 15 min. The broth was neutralized to pH 6.5 and it was also inoculated into wells. The diameter of the zone of inhibition extending laterally around the well was measured and a clear zone of 1 mm or more was considered positive inhibition.

### 2.12. Lysozyme Activity

Evaluation of LAB isolates’ tolerance to lysozyme over 90 min of incubation at 37 °C was carried out as per Ayyash [11]. The bacterial enumeration was carried out on MRS agar using the pour-plate technique after 90 min of incubation at 37 °C. After anaerobic incubation at 37 °C for 48 h, the plates were counted using a colony counter (Interscience San 1200; Park Woburn, MA, USA).

### 2.13. Antibiotic Susceptibility Test

Antibiotic resistance testing was performed according to Shivangi [12] with slight modifications, as MRS and M17 agar plates were used for the respective isolates. The susceptibility of the isolates was tested against penicillin (PEN, 10 mg), clindamycin (CLI, 2 mg), vancomycin (VAN, 30 mg), and erythromycin (ERY, 15 mg). The interpretations of zones as resistant (R), moderately susceptible (MS), and susceptible (S) were assigned according to Charteris [16].

### 2.14. Exopolysaccharide (EPS) Production

EPS production indication testing (−*ve*/+*ve*) was conducted as described by Abushelaibi [14], using milk–ruthenium medium. Overnight cultures were streaked onto the surface of plates containing ruthenium red milk (10% *w*/*v*, skim milk powder, 1% *w*/*v*, sucrose, and 0.08 g/L ruthenium red, 1.5% *w*/*v* agar).

### 2.15. Identification of the LAB Isolates

The 16S rDNA of the selected isolates was amplified according to Alkalbani [17], using PCR primers 27F (5′-AGAGTTTGATCCTGGCTCAG-3′) and 1492R (5′-TACGGYTACCTTGTTACGACTT-3′), and 16S rDNA sequencing of the PCR product was done by Macrogen Sequencing Facilities (Macrogen, Seoul, Korea). The BLAST algorithm in the NCBI database was used to align the sequences and retrieve the accession number for each isolate from the GenBank. An online tool developed by Lemoine [18] was used to determine the bacterial species most closely related to the isolates and to create the dendrogram.

### 2.16. Immunomodulatory Effects

The immunomodulatory effects of the selected isolates were tested using spleen cells of BALB/c and C57BL/6 mice (Jackson Laboratory, Bar Harbor, ME, USA). The immunomodulatory activities of the selected bacterial isolates were tested by incubating them at different concentrations with aseptically prepared single-cell suspensions of murine spleen cells. Splenocytes of BALB/c and C57BL/6 mice were prepared for in vitro culture following a published protocol [19]. Viable red blood cell depleted spleen cells were enumerated based on exclusion of trypan blue dye using a hemocytometer. Viable splenocytes (2 × 10^5^/well) were co-cultured in 96-well plates with either 2 × 10^4^ or 2 × 10^5^ CFUs (equivalent to 10^6^ or 10^7^ CFUs/mL, respectively) of viable or killed LAB isolates (set up in triplicate per group) in RPMI medium supplemented with 10% FBS, penicillin/streptomycin, and gentamicin (Gibco-ThermoFisher Scientific, Waltham, MA, USA). Control wells were also set up in which spleen cells were cultured in medium alone. After 3 days of incubation at 37 °C, cell-free culture supernatants were harvested and kept at −20 °C until they were assayed for cytokines using specific ELISAs, as described previously [20], utilizing commercially available kits for mouse IFN-γ (ThermoFisher Scientific, USA; cat#88-7314) and IL-4 (OptEIA kit; BD Biosciences, USA, ca# 555232). The sensitivity limits of the IFN-γ and IL-4 ELISAs were ~15 pg/mL and ~10 pg/mL, respectively.

### 2.17. Statistical Analysis

One-way ANOVA was applied to determine whether the differences between LAB isolates had a significant influence on the quantitative parameters (*p* < 0.05). Tukey’s test was used to detect differences between mean values with a *p* value of <0.05. To calculate the mean values and standard deviations, all tests were performed at least three times. Minitab version 21.0 (Minitab, Ltd., Coventry, UK) was used for all statistical analyses for noncell line studies. For the immunomodulatory effects, statistical significance between control and LAB isolate-stimulated cultures was analyzed using the unpaired two-tailed Student’s *t*-test. The statistical analyses were performed using GraphPad PRISM 8 software (GraphPad Software, San Diego, CA, USA), and differences with a *p* value ≤ 0.05 were considered significant.

## 3. Results and Discussion

### 3.1. Tolerance to In Vitro Digestive Condition

Table 1 presents the survival rate of LAB isolates after being subjected to in vitro digestion conditions, determined using INFOGEST2.0. After the first step of the in vitro digestion (G0), the average isolate survival ranged from 8.3 to 9.0 Log_10_ CFU/mL. Reductions in the viable numbers of nearly all bacterial isolates were noted particularly after the gastric phase (G120), with different levels depending on the isolate. The survival rate of isolates after G120 ranged from 8.0 to 8.6 Log_10_ CFU/mL. After the intestinal phase (IN-120), the bacterial count ranged from 7.3 to 8.5 Log_10_ CFU/mL, with survival rates higher than ~90% (Table 1).

Probiotics must survive several stresses while in GIT transit, including the low pH of the stomach, bile salts, and digestive enzymes [1]. Thus, at this stage, the isolates with the highest survival rates were selected for further investigations. After IN-120, 29 out of the 46 isolates showed a significant reduction compared to their average in G0, and thus were excluded from further investigations. Only 17 (F1, F5, F8, F13, F15, F18, F21, F23, F25, F26, F28, F31, F37, F40, F41, F43, and F46) out the 46 isolates had remarkable survival rates after the intestinal phase (IN-120), and these were accordingly selected for further assessment. Our results are in accordance with those reported in [21,22]. According to the probiotic definition [3], the probiotic count should be high after transiting the gastric and intestinal conditions in order to confer the expected health benefits. The present results suggest that it is recommend to test the capacity of new LAB isolates to survive GIT conditions in vitro prior to their being employed in in vivo trials (animal or human). It is also recommended to employ a dynamic in vitro digestion system.

### 3.2. Bile Salts Tolerance

The selected 17 isolates were exposed to different bile salts (cholic acid (CA), ox gall (OX), and taurocholic acid (TA)), and their growth percentages are presented in Table 2. Probiotics should possess good resistance toward bile salts in order to survive in the human GIT [1]. Therefore, high survival percentages indicate good bile salt tolerance [21,23]. As shown in Table 2, the survival rates ranged from 22.5 to 53.6%, 32.5 to 51.5%, and 41.8 to 60.9% in MRS supplemented with CA, OX, and TA, respectively, after 3 h of incubation. After 6 h, the survival rates ranged from 17.8 to 51.1%, 33.6 to 63.9%, and 55.9 to 72.5% for CA, OX, and TA, respectively. In other words, the survival rates generally increased against OX and TA and decreased against CA. This implies that CA had more inhibitory effect on the 17 isolates compared with OX and TA. These results are in accordance with those reported by [14].

In short, most isolates had a reasonable resistance to taurocholic acid and oxgall compared to cholic acid. Bile salts have a destructive effect on the membrane lipids of the bacterial cell [1]. Probiotics’ resistance to various bile salts depends on the bacterial species and strains [23,24]. According to previous studies, resistance to bile salts can be due to the presence of polysaccharides on the outer membrane [25].

### 3.3. Bile Salt Hydrolase (BSH) Activities and Cholesterol Removal

Table 3 shows that nearly all isolates had an ability to hydrolyze the bile salt mixture. As shown in Table 3, isolates F25 and F40 had the lowest activity, while isolates F8 and F13 had the highest. In terms of cholesterol removal ability, isolates F1, F8, F23, and F37 had an ability to remove cholesterol from the broth that was more than 30% greater than those of the rest of the isolates (Table 3). BSH activity plays a significant role in inhibiting cholesterol absorption/uptake in the human intestine [1]. The ability to hydrolyze bile salts can disrupt the formation of the cholesterol micelles in the human intestine [1]. For this reason, BSH activity and cholesterol removal are essential tests for probiotic selection [26,27,28]. It has been reported that members of *Enterococcus* spp., *Lactobacillus* spp., *Lacticaseibacillus* spp., and *Streptococcus* spp. isolated from various sources appeared to be similar in their cholesterol reduction effect [29]. Our BSH and cholesterol removal findings agree with those reported by Ayyash [11].

### 3.4. Auto-Aggregation, Hydrophobicity, and Adherence to HT-29 Cells

One of the main criteria required when selecting a probiotic microorganism is its ability to attach to the walls of the intestinal tract [30]. Table 4 presents the results for auto-aggregation, hydrophobicity to three different hydrocarbons (xylene, octane and hexadecane), and attachment to the epithelial cell line (HT-29 cells). In terms of auto-aggregation, after 4 h values ranged from 1.8% to 26.2%, and after 24 h they increased significantly (*p* < 0.05) to between 42.4% and 73.2%, with an average of 59.6%. Auto-aggregation is highly regarded due to its role in forming biofilms which prevent pathogens attachment to the intestine [13,31].

For hydrophobicity, the values were 6.9% to 77.1%, 17.3% to 86.7%, and 29.3% to 84.3% for xylene, octane, and hexadecane, respectively (Table 4). Isolates F26 and F40 had the lowest hydrophobicity for all hydrocarbons. Hydrophobicity to various hydrocarbons is associated with the attachment ability of isolates to epithelial cells. This ability is gained from the hydrophobic components in the outer membrane [13,32].

After allowing isolates to adhere to HT-29 cells, isolates had comparable adherence capabilities with an average of 8.03 Log_10_ CFU/mL after 2 h, while the growth rate ranged from 7.5 to 8.3 Log_10_ CFU/mL (Table 4). These results concur with those reported in [13,15,32] who isolated *Lactobacillus*, *Pediococcus*, and *Lactiplantibacillus* strains from kimchi, alcoholic beverages, and other sources. The adherence ability of probiotics to epithelial cells is considered strain dependent; for this reason, the rate of attachment differs between isolates. Furthermore, the S-layer proteins, flagellin, and cell-bound proteases might affect the attachment aptitude [15,33].

### 3.5. Co-Aggregation and Antimicrobial Activities

The results of co-aggregation against four well-known foodborne pathogens (*E. coli O157:H7*, *S.* Typhimurium, *L. monocytogenes*, and *S. aureus*) after incubation at 37 °C for 4 and 24 h are presented in Table 5. After 2 h, the co-aggregation rates ranged from ~3.44 to 10.43%, and the range (~21.38 to 42.61%) rose after 24 h. This indicates that co-aggregation ability is directly correlated to time [14]. Isolates F31 and F41 had the highest and lowest co-aggregation rates, respectively, with all pathogens after 24 h (Table 5). Analysis of variance did not exhibit any significant difference in co-aggregation between the four foodborne pathogens given the same isolate and same time. As noted in Table 6, postbiotics, which are heat-killed bacteria, had better antimicrobial effects against *E. coli* than probiotics, while probiotics had better effects against *S. aureus* and *L. monocytogenes* when compared with postbiotics. It has been reported that metabolites produced by LAB isolates such as bacteriocins, peptides, organic acids, and volatile compounds are highly associated with antimicrobial activity [14,21,32]. The antimicrobial activity of the killed cells suggests that the cell membrane and cytoplasm possess antimicrobial activities against foodborne pathogens. Our results coincide with those reported by [5].

### 3.6. Lysozyme Tolerance, Antibiotic Resistance, and EPS Production

Lysozyme is an enzyme present specifically in the saliva. It has antimicrobial activity, and thus acts as the first barrier in the mouth [34]. Therefore, tolerance against this enzyme is required for an isolate to be a good probiotic. All isolates exhibited high tolerance to lysozyme; the average growth at the start time and after 90 min was ~8.31 Log_10_ CFU/mL (Table 7). This outcome agrees with the results obtained for isolates from sausages, camel milk, fermented foods, and pickles [14,17,35].

Table 7 presents the results of the antibiotic resistance test against four different antibiotics, namely vancomycin, erythromycin, penicillin, and clindamycin. All isolates generally had resistance against vancomycin. This may be attributed to the natural resistance against glycopeptides, such as vancomycin, which was caused by membrane impermeability [21,29]. On the other hand, all isolates were susceptible or sensitive to the rest of the antibiotics.

The results of exopolysaccharide production (EPS) are summarized in Table 7. While isolates F1, F5, F21, F23, F25, F26, F43, and F46 did not show an ability to produce EPS, the rest of the isolates were able to form EPS. EPS are produced in bacteria either freely in the medium or in the form of capsules. EPSs are significantly correlated with biofilm formation, attachment to the intestinal cell wall, and cholesterol reduction, and provide protection against an harsh environment [36].

### 3.7. Identification by 16S rRNA

**Figure 1:**
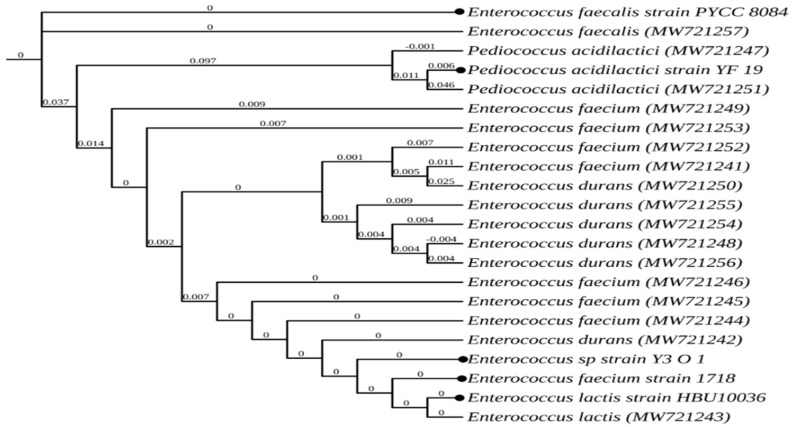
Polygenetic tree based on 16S rRNA sequences. Numbers in parentheses are accession numbers of identified sequences from GenBank. Filled circles are the reference strains from NCBI.

All 17 isolates were identified using 16S rRNA, aligned, and divided into four groups of LAB, and are listed with their accession numbers in Table 8. A polygenetic tree was created using the online tool shown in Figure 1, which is ‘https://ngphylogeny.fr/’, in order to determine LAB species level, which was based on the 16S rRNA sequences via the neighbor-joining method [18].

### 3.8. Immunomodulatory Effect

The interaction between gut microbiota and the immune system is well described in the literature. In several studies, probiotics have been shown to have anti-allergic properties, primarily via the induction of a predominant Th1 cytokine response [37,38,39]. One of these studies claimed that probiotics can reduce allergy-induced damage to the host and identified three LABs with these properties [38]. Based on our results, two potential probiotics (isolates F8 and F28) were selected for further immunological investigation.

**Figure 2:**
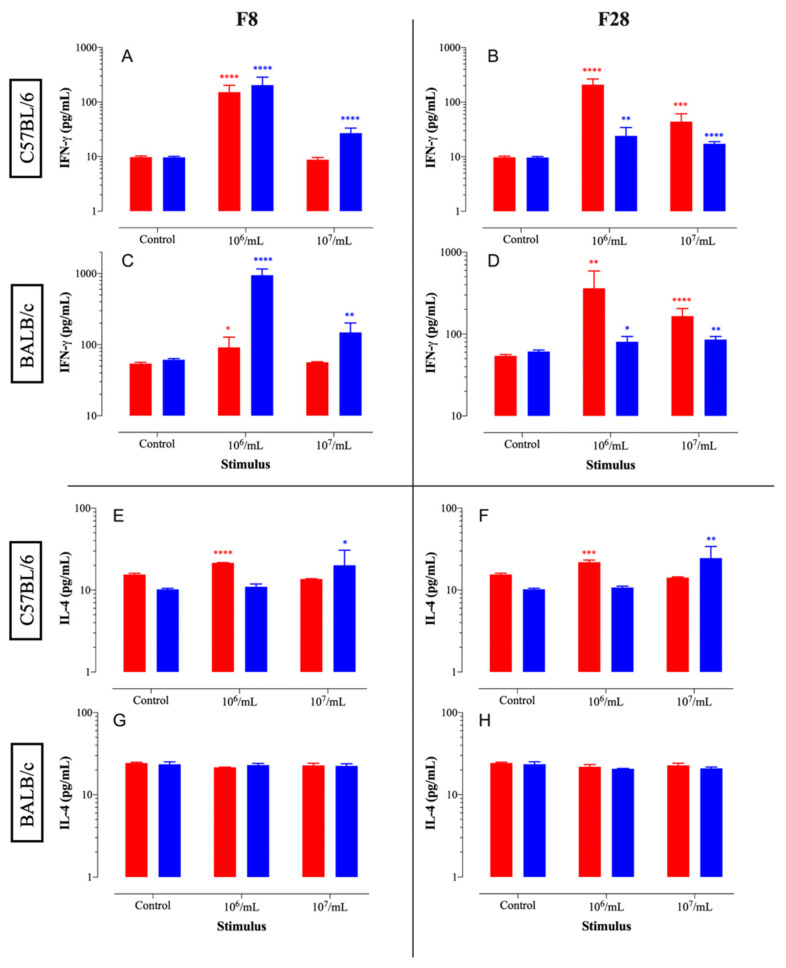
Immunomodulatory effects of two potential probiotics (live, red bar) and postbiotics (killed, blue bar) of F8 (**A**,**C**,**E**,**G**) and F28 (**B**,**D**,**F**,**H**) isolates against spleen cells C57BL/6 and BALB/c. (**A**,**B**) IFN-γ response of C57BL/6, (**E**,**F**) IFN-γ response of BALB/c, (**C**,**D**) IL-4 response of C57BL/6, (**G**,**H**) IL-4 response of BALB/c against MBL3 and MBL10. Asterisks denote statistically significant differences between the indicated groups and the corresponding control groups (* *p* < 0.05; ** *p* < 0.01; *** *p* < 0.001; **** *p* < 0.0001).

The immunomodulatory properties of isolates F8 and F28 as probiotics (live bacteria) and postbiotics (killed bacteria) were assessed via their ability to induce the secretion of IFN-γ and IL-4 cytokines in ex vivo cultured murine spleen cells. The studies were carried out using spleen cells obtained from two genetically different inbred mouse strains, namely C57BL/6 and BALB/c mice (Figure 2). This was done in order to ensure that the immunological profiles of the LAB strains were applicable across different host genetic backgrounds [40]. The results demonstrate that LAB isolates F8 and F28 induced the production of IFN-γ by spleen cells (Figure 2A–D). The detectable IFN-γ levels were higher when isolates F8 and F28 were used at the 10^6^/mL concentration, most likely reflecting the fact that the higher LAB concentration of 10^7^/mL was toxic to splenocytes. Furthermore, the isolate F8 induced significantly higher IFN-γ secretion when used as a postbiotic (killed preparation) than q probiotic (live bacteria) (Figure 2A,C). In contrast, isolate F28 reproducibly induced higher IFN-γ levels when used as a probiotic, regardless of the mouse strain (Figure 2B,D). Moreover, no appreciable induction of IL-4 secretion was evident in cultures of spleen cells with either LAB isolate (Figure 2E–H). Interestingly, both isolates F8 and F28 appeared to induce higher IFN-γ levels by BALB/c splenocytes than C57BL/6 cells, highlighting the strong pro Th1-inducing capacity of both isolates. This is striking given the known propensity of BALB/c mice to develop Th2 immune responses [40]. These findings confirm the capacity of LAB isolates to induce IFN-γ from in vitro cultured spleen cells, suggesting their potential to antagonize Th2 responses in vivo. Further experiments are needed to confirm these findings in a preclinical allergy model.

## 4. Conclusions

To sum up, in vitro digestion was performed to screen for good probiotic characteristics involved in bile and lysozyme tolerance, cholesterol reduction activity, attachment property including hydrophobicity to hydrocarbons, auto-aggregation, co-aggregation, and attachment to HT-29 cells. Moreover, the selected isolates displayed antimicrobial and salt hydrolysis activities, and antibiotic susceptibility. Isolates F1, F5, F8, F15, F18, F21, F23, F25, F26, F28, F40, F43, and F46 displayed potential probiotic and postbiotic properties. However, F8 and F28 exhibited noticeable immunomodulatory activities. The immunomodulatory activities suggest a promising role of these isolates if used in animal studies and later human trials. Moreover, EPS was produced by all isolates except isolates F1, F5, F21, F23, F25, F26, F43, and F46 (*Enterococcus faecium*, *E. durans*, *E. lactis*, and *Pediococcus acidilactici*) which can be used to characterize isolates for industrial and medical purposes.

## Figures and Tables

**Table 1:** In vitro digestive system tolerance (Log_10_ CFU/mL) of lactic acid bacteria isolated from fresh vegetables.

Isolates	Starting	After Gastric	After Intestine
F1	8.8 ± 0.42	8.3 ± 0.67	8.2 ± 0.59
F2	8.6 ± 0.26	8.5 ± 0.76	8.2 ± 0.61
F3	8.6 ± 0.28	8.4 ± 0.77	8.1 ± 0.50
F4	8.8 ± 0.52	8.5 ± 0.85	8.3 ± 0.70
F5	8.9 ± 0.45	8.5 ± 0.85	8.1 ± 0.70
F6	8.8 ± 0.37	8.4 ± 0.72	8.2 ± 0.53
F7	8.8 ± 0.49	8.4 ± 0.84	8.3 ± 0.73
F8	8.9 ± 0.44	8.4 ± 0.79	8.3 ± 0.65
F9	8.9 ± 0.54	8.2 ± 0.90	8.3 ± 0.75
F10	8.7 ± 0.44	8.4 ± 0.78	8.4 ± 0.73
F11	8.8 ± 0.57	8.1 ± 0.87	8.3 ± 0.67
F12	8.7 ± 0.53	8.5 ± 0.86	8.4 ± 0.69
F13	8.7 ± 0.41	8.4 ± 0.63	8.3 ± 0.74
F14	8.7 ± 0.43	8.4 ± 0.77	8.2 ± 0.78
F15	8.8 ± 0.38	8.4 ± 0.72	8.5 ± 0.75
F16	8.8 ± 0.31	8.4 ± 0.77	8.4 ± 0.68
F17	8.8 ± 0.30	8.4 ± 0.69	8.3 ± 0.73
F18	8.9 ± 0.46	8.4 ± 0.71	8.4 ± 0.75
F19	8.3 ± 0.09	8.1 ± 0.62	7.9 ± 0.58
F20	8.4 ± 0.18	8.3 ± 0.68	7.6 ± 0.57
F21	8.9 ± 0.49	8.6 ± 0.83	8.4 ± 0.66
F22	8.4 ± 0.32	8.3 ± 0.61	8.3 ± 0.64
F23	8.6 ± 0.31	8.5 ± 0.88	8.2 ± 0.61
F24	8.8 ± 0.46	8.4 ± 0.77	7.9 ± 0.35
F25	8.7 ± 0.39	8.3 ± 0.78	8.2 ± 0.59
F26	8.8 ± 0.53	8.5 ± 0.87	8.1 ± 0.47
F27	8.4 ± 0.68	8.4 ± 0.85	8.1 ± 0.50
F28	9.0 ± 0.57	8.5 ± 0.93	8.4 ± 0.81
F29	8.8 ± 0.48	8.4 ± 0.90	7.6 ± 0.38
F30	8.8 ± 0.51	8.6 ± 1.00	8.1 ± 0.51
F31	8.7 ± 0.54	8.4 ± 0.78	8.2 ± 0.50
F32	8.9 ± 0.61	8.2 ± 0.63	7.9 ± 0.44
F33	8.6 ± 0.57	8.4 ± 0.74	7.7 ± 0.46
F34	8.3 ± 0.48	8.0 ± 0.69	8.0 ± 0.40
F35	8.6 ± 0.50	8.2 ± 0.59	7.9 ± 0.37
F36	8.8 ± 0.43	8.4 ± 0.68	7.7 ± 0.30
F37	8.9 ± 0.52	8.4 ± 0.84	8.2 ± 0.50
F38	8.7 ± 0.40	8.3 ± 0.66	7.4 ± 0.20
F39	8.8 ± 0.54	8.2 ± 0.71	8.1 ± 0.56
F40	8.8 ± 0.58	8.5 ± 0.81	8.2 ± 0.52
F41	8.9 ± 0.57	8.4 ± 0.72	8.5 ± 0.85
F42	8.8 ± 0.51	8.5 ± 0.93	8.1 ± 0.53
F43	8.9 ± 0.78	8.5 ± 0.79	8.5 ± 0.74
F44	8.8 ± 0.52	8.5 ± 0.87	8.1 ± 0.53
F45	9.0 ± 0.52	8.4 ± 0.72	8.4 ± 0.62
F46	8.6 ± 0.38	8.4 ± 0.71	8.2 ± 0.49

Values are expressed as the mean ± standard deviation of triplicates.

**Table 2:** Bile tolerances (%) for 17 selected lactic acid bacteria isolated from fresh vegetables.

Isolates	3 h			6 h		
	CA	OX	TA	CA	OX	TA
F1	43.9	42.7	56.1	39.3	39.9	58.8
F5	29.1	38.9	48.7	27.6	52.4	63.0
F8	53.7	40.2	60.9	51.1	39.0	60.7
F13	46.2	37.4	59.5	45.3	33.6	60.0
F15	45.0	39.9	59.0	41.5	35.2	57.0
F18	41.4	40.7	60.0	36.4	38.8	58.1
F21	22.5	39.7	49.8	20.5	51.1	63.8
F23	26.7	33.5	44.9	23.9	49.9	59.6
F25	32.3	40.7	55.3	35.4	55.7	66.9
F26	33.0	42.5	41.8	29.7	60.6	56.0
F28	26.3	42.1	51.2	17.8	56.0	63.5
F31	28.0	35.6	47.5	25.2	52.4	64.0
F37	33.0	32.5	45.4	37.3	55.8	62.7
F40	36.1	50.1	48.3	34.5	62.2	62.2
F41	35.3	40.7	45.5	29.3	42.9	63.6
F43	32.3	45.1	56.3	37.8	50.4	72.5
F46	37.6	51.5	54.8	44.4	63.9	68.6

Values are expressed as the mean ± standard deviation of triplicates. CA = cholic acid, OX = oxgall, TA = taurocholic acid.

**Table 3:** Bile salt hydrolytic activity (BSH; U/mg) and cholesterol removal (CR; %) ability of lactic acid bacteria isolated from fresh vegetables.

Isolates	BSH (U/mg)	CR (%)
F1	0.83 ± 0.03 ^f^	35.0 ± 1.41 ^a^
F5	0.95 ± 0.05 ^b^	27.5 ± 2.12 ^e^
F8	1.03 ± 0.07 ^a^	34.5 ± 0.71 ^a^
F13	1.00 ± 0.08 ^a^	17.5 ± 0.71 ^g^
F15	0.93 ± 0.05 ^b^	17.0 ± 1.41 ^g^
F18	0.83 ± 0.03 ^f^	29.5 ± 0.71 ^d^
F21	0.91 ± 0.06 ^c^	30.0 ± 0.23 ^c^
F23	0.87 ± 0.07 ^d^	33.0 ± 1.41 ^b^
F25	0.80 ± 0.02 ^g^	30.5 ± 3.54 ^c^
F26	0.84 ± 0.04 ^e^	24.5 ± 2.12 ^f^
F28	0.84 ± 0.04 ^e^	29.0 ± 0.99 ^d^
F31	0.84 ± 0.05 ^e^	30.0 ± 2.83 ^c^
F37	0.82 ± 0.04 ^f^	35.0 ± 0.98 ^a^
F40	0.80 ± 0.03 ^g^	30.0 ± 2.83 ^c^
F41	0.81 ± 0.06 ^g^	27.5 ± 2.12 ^e^
F43	0.97 ± 0.04 ^a,b^	24.5 ± 2.12 ^f^
F46	0.91 ± 0.07 ^c^	30.0 ± 2.83 ^c^

Values are the mean ± standard deviation of triplicates. ^a–g^ Mean values in the same column with different lowercases differ significantly (*p* < 0.05).

**Table 4:** Auto-aggregation (%), hydrophobicity (%), and attachment to HT-29 cells (Log_10_ CFU/well) of potential probiotic lactic acid bacteria isolated from fresh vegetables.

Isolates	Auto-Aggregation (%)		Hydrophobicity (%)			Attachment to HT-29 Cells
	4 h	24 h	Xylene	Octane	Hexadecane	Log_10_ CFU
F1	26.1 ± 0.87 ^a^	66.5 ± 2.83 ^c^	61.4 ± 3.07 ^b^	68.7 ± 2.06 ^c^	76.1 ± 3.05 ^b^	8.0 ± 0.08 ^c^
F5	9.8 ± 2.27 ^d^	63.6 ± 2.57 ^d^	35.9 ± 1.79 ^c,d^	40.7 ± 1.22 ^d,e^	47.3 ± 1.89 ^c^	8.0 ± 0.00 ^c^
F8	15.6 ± 1.90 ^c^	45.0 ± 2.19 ^f^	77.1 ± 3.86 ^a^	79.0 ± 2.37 ^b^	84.3 ± 3.37 ^a^	8.1 ± 0.02 ^b^
F13	26.2 ± 0.02 ^a^	56.7 ± 0.13 ^e^	71.0 ± 3.55 ^a^	86.7 ± 2.60 ^a^	82.0 ± 3.28 ^a,b^	8.0 ± 0.10 ^c^
F15	18.9 ± 0.31 ^b^	47.2 ± 0.60 ^f^	66.0 ± 3.30 ^b^	79.0 ± 2.37 ^b^	80.7 ± 3.23 ^b^	8.1 ± 0.07 ^b^
F18	17.4 ± 0.58 ^b^	42.9 ± 0.29 ^g^	56.9 ± 2.84 ^b,c^	69.4 ± 2.08 ^c^	73.3 ± 2.93 ^b,c^	8.1 ± 0.04 ^b^
F21	2.0 ± 0.16 ^h^	70.3 ± 0.84 ^b^	13.3 ± 0.66 ^f^	36.6 ± 1.10 ^d,e^	39.6 ± 1.58 ^d^	8.1 ± 0.04 ^b^
F23	3.8 ± 1.15 ^f^	73.2 ± 2.90 ^a^	32.7 ± 1.64 ^d^	38.3 ± 1.15 ^d,e^	45.7 ± 1.83 ^c^	8.1 ± 0.03 ^b^
F25	2.3 ± 1.06 ^g^	63.6 ± 1.44 ^d^	13.7 ± 0.69 ^f^	28.6 ± 0.86 ^e^	32.1 ± 1.29 ^e^	8.1 ± 0.04 ^b^
F26	3.6 ± 0.30 ^f^	70.6 ± 2.61 ^b^	13.0 ± 0.65 ^f^	17.3 ± 0.52 ^g^	29.3 ± 1.17 ^f^	7.9 ± 0.03 ^d^
F28	1.8 ± 0.13 ^h^	70.5 ± 1.99 ^b^	11.4 ± 0.57 ^g^	29.9 ± 0.90 ^e^	34.9 ± 1.39 ^d,e^	8.0 ± 0.07 ^c^
F31	2.4 ± 0.16 ^g^	72.0 ± 1.17 ^a^	18.9 ± 0.94 ^e^	23.7 ± 0.71 ^e,f^	42.3 ± 1.69 ^c^	8.1 ± 0.06 ^b^
F37	8.2 ± 0.34 ^d^	70.5 ± 2.73 ^b^	33.4 ± 1.67 ^c,d^	21.7 ± 0.65 ^f^	34.1 ± 1.37 ^d,e^	8.0 ± 0.05 ^c^
F40	8.0 ± 0.53 ^e^	63.4 ± 1.02 ^d^	6.9 ± 0.34 ^h^	30.3 ± 0.91 ^e^	35.3 ± 1.41 ^d,e^	7.5 ± 0.01 ^d^
F41	11.1 ± 1.04 ^c^	35.2 ± 0.35 ^h^	34.0 ± 1.70 ^c,d^	44.6 ± 1.34 ^d^	30.7 ± 1.23 ^e^	8.0 ± 0.05 ^c^
F43	9.4 ± 1.91 ^d^	42.4 ± 1.61 ^g^	40.1 ± 2.01 ^c^	56.1 ± 1.68 ^c,d^	46.9 ± 1.87 ^c^	8.1 ± 0.02 ^b^
F46	-	-	31.0 ± 1.55 ^d^	45.7 ± 1.37 ^d^	39.9 ± 1.59 ^d^	8.3 ± 0.08 ^a^

Values are the mean ± standard deviation of triplicates. ^a–h^ Mean values in the same column with different lowercases differ significantly (*p* < 0.05).

**Table 5:** Co-aggregation (%) of potential probiotic lactic acid bacterial isolates with four pathogenic bacteria.

Isolates	2 h				24 h			
	*E. coli*	*S. typhi*	*S. aureus*	*L. monocytogenes*	*E. coli*	*S. typhi*	*S. aureus*	*L. monocytogenes*
F1	10.1 ± 0.50 ^a^	10.6 ± 0.63 ^a^	10.0 ± 0.70 ^a^	10.2 ± 0.71 ^a^	25.5 ± 1.78 ^d^	32.7 ± 1.31 ^b^	34.8 ± 0.70 ^d^	32.6 ± 1.63 ^b^
F5	7.6 ± 0.38 ^c^	8.4 ± 0.51 ^b,c^	9.7 ± 0.68 ^b^	8.0 ± 0.56 ^b^	23.8 ± 1.67 ^d^	24.4 ± 0.98 ^c^	27.1 ± 0.54 ^e^	25.0 ± 1.25 ^d,e^
F8	9.4 ± 0.47 ^a,b^	6.7 ± 0.40 ^d^	10.0 ± 0.70 ^a^	6.8 ± 0.47 ^d^	21.7 ± 1.52 ^e^	23.8 ± 0.95 ^c,d^	27.4 ± 0.55 ^e^	23.9 ± 1.19 ^d,e^
F13	6.8 ± 0.34 ^e,d^	9.6 ± 0.58 ^b^	9.3 ± 0.65 ^b^	10.3 ± 0.72^a^	22.1 ± 1.55 ^e^	28.0 ± 1.12 ^b,c^	31.3 ± 0.63 ^d^	28.6 ± 1.43 ^c^
F15	8.4 ± 0.42 ^b^	9.4 ± 0.57 ^b^	10.8 ± 0.76 ^a^	8.7 ± 0.61 ^b^	22.4 ± 1.57 ^e^	26.7 ± 1.07 ^c^	30.7 ± 0.61 ^d^	26.2 ± 1.31 ^d^
F18	7.8 ± 0.39 ^c^	5.9 ± 0.35 ^e^	8.2 ± 0.58 ^c^	6.7 ± 0.47 ^d^	20.5 ± 1.44 ^e^	22.5 ± 0.90 ^d^	29.7 ± 0.59 ^e^	24.8 ± 1.24d ^e^
F21	5.5 ± 0.27 ^d^	3.8 ± 0.23 ^g^	4.4 ± 0.31 ^d^	3.9 ± 0.27 ^f^	38.6 ± 2.70 ^b^	40.0 ± 1.60	38.6 ± 0.77 ^b^	35.6 ± 1.78 ^b^
F23	4.0 ± 0.20 ^e^	6.7 ± 0.40 ^d^	6.3 ± 0.44 ^de^	6.1 ± 0.43 ^d^	43.8 ± 3.07 ^a^	40.8 ± 1.63 ^b^	39.2 ± 0.78 ^b^	28.0 ± 1.40 ^c^
F25	4.1 ± 0.21 ^e^	5.5 ± 0.33 ^e^	5.1 ± 0.36 ^e^	3.8 ± 0.27 ^f^	33.7 ± 2.36 ^c^	42.3 ± 1.69 ^a^	44.0 ± 0.88 ^a^	36.6 ± 1.83 ^a,b^
F26	4.9 ± 0.25 ^e^	7.7 ± 0.46 ^c^	4.9 ± 0.34 ^f^	6.2 ± 0.43 ^d^	43.5 ± 3.05 ^a^	39.1 ± 1.56 ^a,b^	39.4 ± 0.79 ^b^	38.7 ± 1.94 ^a^
F28	3.2 ± 0.16 ^f^	4.4 ± 0.26 ^f^	3.2 ± 0.23 ^g^	5.1 ± 0.36 ^e^	42.4 ± 2.97 ^a^	33.8 ± 1.35 ^b^	37.4 ± 0.75 ^c^	31.8 ± 1.59 ^b^
F31	2.9 ± 0.15 ^g^	5.5 ± 0.33 ^e^	4.7 ± 0.33 ^g^	3.9 ± 0.27 ^f^	45.5 ± 3.18 ^a^	40.7 ± 1.63 ^a,b^	40.7 ± 0.81 ^b^	36.7 ± 1.83 ^a,b^
F37	3.4 ± 0.17 ^f^	6.1 ± 0.37 ^d^	9.1 ± 0.64 ^b^	5.7 ± 0.40 ^e^	30.5 ± 2.14 ^c^	24.2 ± 0.97 ^c^	27.4 ± 0.55	26.4 ± 1.32 ^d^
F40	5.7 ± 0.28 ^d^	7.7 ± 0.46 ^c^	7.1 ± 0.50 ^c,d^	6.7 ± 0.47 ^d^	26.6 ± 1.86 ^d^	25.7 ± 1.03 ^c^	28.5 ± 0.57 ^e^	26.5 ± 1.32 ^d^
F41	5.7 ± 0.28 ^d^	6.4 ± 0.38 ^d^	7.5 ± 0.53 ^c,d^	7.1 ± 0.49 ^c^	18.2 ± 1.28 ^f^	21.0 ± 0.84 ^d^	25.2 ± 0.50 ^f^	21.2 ± 1.06 ^e^
F43	5.1 ± 0.26 ^de^	5.2 ± 0.31 ^e^	7.0 ± 0.49 ^d^	6.1 ± 0.43 ^d^	21.5 ± 1.50 ^e^	22.7 ± 0.91 ^d^	27.8 ± 0.56 ^e^	21.1 ± 1.05 ^e^
F46								

Values are the mean ± standard deviation of triplicates. ^a–g^ Mean values in the same column with different lowercases differ significantly (*p* < 0.05).

**Table 6:** Antimicrobial activities of potential probiotic and postbiotic lactic acid bacterial isolates against four foodborne pathogenic bacteria.

Isolate	Probiotic ^a^				Postbiotic ^b^			
	*E. coli*	*S. aureus*	*S. typhi*	*L. monocytogenes*	*E. coli*	*S. aureus*	*S. typhi*	*L. monocytogenes*
F1	+	++	+++	+++	+++	+++	+++	+++
F5	+	++	++	+++	++	++	++	+++
F8	+	++	++	++	++	++	++	++
F13	+	+++	+++	+++	++	++	++	++
F15	+	+++	+++	+++	+	++	++	+++
F18	+	++	+++	+++	++	++	++	++
F21	+	+++	+++	+++	++	++	++	++
F23	+	+++	+++	++	++	+	++	+++
F25	+	++	++	++	++	+++	++	++
F26	+	++	++	++	++	++	++	+++
F28	+	++	++	+++	++	++	++	++
F31	+	+++	++	++	++	++	++	+++
F37	+	++	++	++	+	++	++	++
F40	+	+	+	+	+	+	+	+
F41	+	++	+	+	+	+	+	+
F43	+	+	++	+	+	+	+	+
F46	+	++	++	+	+	+	+	+

(+) log reduction was <1.0; (++) log reduction was less than 2.0 and higher than 1.0; (+++) log reduction was greater than 2.1. ^a^ Probiotic: live bacteria. ^b^ Postbiotic: heat-killed bacteria.

**Table 7:** Lysozyme tolerance (Log_10_ CFU/mL), antibiotic susceptibility, and exopolysaccharide (EPS) production of lactic acid bacteria isolated from fresh vegetables.

Isolate	Lysozyme Tolerance		Antibiotic Susceptibility				EPS Production
	0 min	90 min	Vancomycin	Erythromycin	Penicillin	Clindamycin	
F1	8.1 ± 0.47 ^c^	8.3 ± 0.27 ^b,c^	R	S	S	S	-
F5	8.1 ± 0.45 ^c^	8.1 ± 0.04 ^d^	R	S	S	S	-
F8	8.8 ± 0.57 ^a^	8.4 ± 0.49 ^b^	R	R	S	R	+
F13	8.5 ± 0.49 ^b^	8.2 ± 0.25 ^c^	R	MS	S	R	+
F15	8.5 ± 0.31 ^b^	8.3 ± 0.34 ^b,c^	R	S	S	MS	+
F18	8.1 ± 0.35 ^c^	8.2 ± 0.32 ^c^	R	S	S	R	+
F21	8.0 ± 0.43 ^c^	8.2 ± 0.27 ^c^	R	S	S	S	-
F23	8.8 ± 0.36 ^a^	8.2 ± 0.25 ^c^	R	S	S	S	-
F25	8.1 ± 0.59 ^c^	8.3 ± 0.25 ^b,c^	R	S	S	S	-
F26	8.8 ± 0.40 ^a^	8.4 ± 0.32 ^b^	R	S	S	S	-
F28	8.0 ± 0.52 ^c^	8.5 ± 0.47 ^a^	R	S	S	S	+
F31	8.6 ± 0.27 ^a,b^	8.2 ± 0.23 ^c^	R	MS	S	S	+
F37	8.4 ± 0.33 ^b,c^	8.3 ± 0.29 ^b,c^	R	MS	MS	MS	+
F40	8.4 ± 0.32 ^b,c^	8.4 ± 0.42 ^b^	R	S	S	S	+
F41	8.4 ± 0.33 ^b,c^	8.4 ± 0.31 ^b^	R	S	S	MS	+
F43	8.5 ± 0.46 ^b^	8.3 ± 0.35 ^b,c^	R	S	S	S	-
F46	8.4 ± 0.37 ^b,c^	8.6 ± 0.45 ^a^	R	S	S	S	-

R = resistant; MS = moderately resistant; S = susceptible. ^a–d^ Mean values in the same column with different lowercases differ significantly (*p* < 0.05).

**Table 8:** Identification of lactic acid bacterial isolates using 16S rRNA gene sequencing and their accession numbers from GenBank.

Sample	Organism	Accession No
F1	*Enterococcus faecium*	MW721241
F5	*Enterococcus durans*	MW721242
F8	*Enterococcus lactis*	MW721243
F13	*Enterococcus faecium*	MW721244
F15	*Enterococcus faecium*	MW721245
F18	*Enterococcus faecium*	MW721246
F21	*Pediococcus acidilactici*	MW721247
F23	*Enterococcus durans*	MW721248
F25	*Enterococcus faecium*	MW721249
F26	*Enterococcus durans*	MW721250
F28	*Pediococcus acidilactici*	MW721251
F31	*Enterococcus faecium*	MW721252
F37	*Enterococcus faecium*	MW721253
F40	*Enterococcus durans*	MW721254
F41	*Enterococcus durans*	MW721255
F43	*Enterococcus durans*	MW721256
F46	*Enterococcus faecalis*	MW721257

## Data Availability

Not applicable.
